# Senescence-Associated Transcriptome Reversal Prioritizes Repurposing Candidates for Diabetic Kidney Disease: A Computational Study

**DOI:** 10.3390/biomedicines14102286

**Published:** 2026-10-09

**Authors:** Duygu Aygüneş-Jafari, Zekeriya Düzgün

**Affiliations:** 1 Department of Medical Biology, Faculty of Medicine, Ege University, 35100 Izmir, Turkey; 2 Department of Medical Biology, Faculty of Medicine, Giresun University, 28200 Giresun, Turkey; zekeriya.duzgun@giresun.edu.tr

**Keywords:** diabetic kidney disease, cellular senescence, metabolic ageing, drug repurposing, senotherapeutics, transcriptome reversal, single-nucleus transcriptomics

## Abstract

**Background/Objectives:** Diabetic kidney disease (DKD) is a leading cause of end-stage kidney disease, and cellular senescence is a tractable therapeutic target; yet prioritizing credible repurposing candidates is difficult because bulk-tissue senescence signals are weak, single-dataset analyses are unstable, and transcriptome-reversal screens overstate novelty. The objective was to build a senescence-anchored, multi-evidence computational prioritization of repurposing candidates for DKD entirely from open data. **Methods:** Per-sample senescence activity was quantified by single-sample gene set enrichment across three DKD kidney cohorts, and consensus signatures were reversed against 45,956 LINCS L1000 signatures. Candidates were ranked by reversal strength, mechanism, ChEMBL stage, and ClinicalTrials.gov novelty, then assessed for robustness by senotherapeutic enrichment, an external senolytic benchmark, leave-one-cohort-out analysis, drug–target Mendelian randomization, and single-nucleus profiling. **Results:** Senescence activity was consistently elevated in DKD across all three cohorts, with moderate-to-large effect sizes (rank-biserial 0.32–0.78). Reversal recovered established senotherapeutic classes and nominated the approved JAK inhibitor fedratinib (within the same class, baricitinib reduced albuminuria in a Phase 2 DKD trial); curated senolytics were modestly separated from negatives (AUROC 0.62, 95% CI 0.52–0.72). Filtering yielded 867 mechanism-annotated compounds robust to cohort removal. Drug–target Mendelian randomization was inconclusive, and single-nucleus profiling highlighted endothelial activation rather than a stable senescent compartment. **Conclusions:** The result is an externally benchmarked, novelty-annotated senotherapeutic candidate map, offered as convergent confirmation of repurposable mechanisms rather than a claim of new drug classes.

## 1. Introduction

Diabetic kidney disease develops in approximately 40% of people with diabetes and is the leading cause of chronic kidney disease and end-stage kidney disease worldwide. Despite renin–angiotensin blockade, sodium–glucose cotransporter-2 inhibition and non-steroidal mineralocorticoid-receptor antagonism, a large residual risk of progression remains [1]. New mechanisms of intervention are therefore needed, and the most readily translatable are agents with an established human safety profile that can be repurposed.

Cellular senescence has emerged as one such mechanism. Senescent cells accumulate in the diabetic kidney, where the senescence-associated secretory phenotype (SASP) sustains inflammation and fibrosis, and their clearance or modulation is increasingly viewed as a therapeutic objective [2,3,4,5]. In this respect, DKD can be regarded as a disorder of accelerated tissue ageing, in which programs that accumulate with chronological age in the kidney, chiefly cellular senescence and its secretory phenotype, are engaged prematurely under sustained metabolic stress [2,5]. Framing the disease in these terms makes the mechanisms of biological ageing, rather than glycemic and haemodynamic control alone, a legitimate target for pharmacological intervention. Bioinformatic work has established that senescence-associated transcriptional activity is detectable in DKD kidney tissue [6], and the broader senotherapeutic field has begun to apply gene-expression similarity and machine learning to nominate senolytic and senomorphic compounds [7,8,9,10]. The two classes differ in mechanism and in how they would be used: senolytics eliminate senescent cells by transiently disabling senescent-cell anti-apoptotic pathways, which permits intermittent dosing, whereas senomorphics do not kill senescent cells but suppress the SASP and therefore require continuous exposure [11]; senotherapeutics is the umbrella term for both. In DKD, the senolytic combination of dasatinib and quercetin has reached an open-label pilot trial [12]. A disease-anchored link remains absent: a prioritization that starts from a DKD senescence-associated transcriptional signature, reverses it against a perturbation atlas, and is then subjected to the validation steps that distinguish a credible prioritized set from a list of nominally plausible compounds.

Three problems recur in this class of study and shape the present design. First, senescence signals in bulk kidney tissue are weak because senescent cells are a minority population; a single cohort may yield misleading results, and the choice of enrichment statistic influences the outcome. Second, transcriptome reversal recovers senomorphic as readily as senolytic activity; the appropriate benchmark is therefore the broader senotherapeutic category rather than apoptosis-inducing senolytics alone. Third, the highest-ranked candidates frequently represent mechanisms that the field has already explored, and a rigorous analysis should distinguish convergent confirmation from genuine novelty.

The present study addresses these problems directly. Senescence-associated transcriptional activity is quantified per sample across three distinct DKD cohorts; reversal is computed against the complete 45,956-signature reference packaged for signatureSearch rather than a truncated list of top-ranked signatures; the ranking is benchmarked against an external, independently curated senolytic resource [9]; candidate mechanisms are cross-checked against the existing DKD and clinical-trial literature; and the bulk-tissue result is examined at cellular resolution through single-nucleus RNA sequencing. The result is a prioritized, development-stage- and novelty-annotated map of candidate senotherapeutic mechanisms and compounds for DKD, presented with calibrated claims about which findings are novel and which are confirmatory.

## 2. Materials and Methods

### 2.1. Data Sources

**Figure 1:**
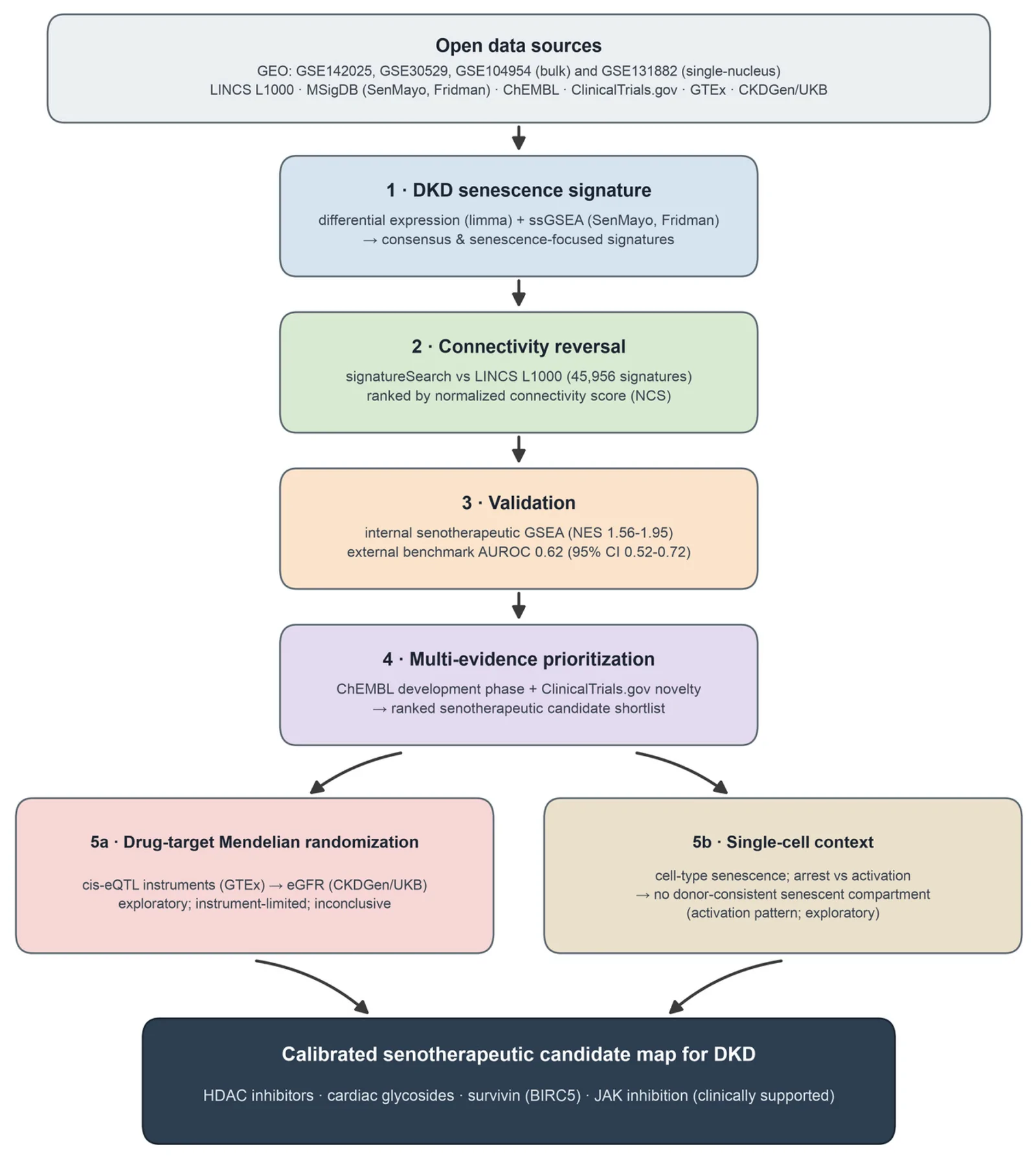
Study workflow. Overview of the senescence-associated drug-repurposing pipeline for diabetic kidney disease (DKD). Open transcriptomic data from three bulk DKD cohorts (GSE142025, GSE30529, GSE104954) and one single-nucleus dataset (GSE131882) were used to quantify per-sample senescence-associated transcriptional activity (ssGSEA) and derive consensus and senescence-focused signatures, which were reversed against the LINCS L1000 atlas (signatureSearch) and prioritized by reversal strength, mechanism of action, ChEMBL development stage and ClinicalTrials.gov novelty. The ranking was benchmarked against an independent senolytic resource, tested for robustness (leave-one-cohort-out and up-regulated-genes-only), probed by exploratory drug–target Mendelian randomization, and contextualized at single-nucleus resolution. Arrows indicate the direction of the analysis workflow.

The overall analysis workflow is summarized in Figure 1. All data were obtained from open repositories and used in de-identified, processed form; no controlled-access data were used. The analyzed cohorts are summarized in Table 1. Three human kidney transcriptomic cohorts with DKD and control groups were analyzed: GSE142025 (bulk RNA-seq; 27 DKD expression profiles available in the deposited series and 9 controls; the source study reports 28 enrolled DKD patients) [13], GSE30529 (tubulointerstitial microarray; 10 diabetic and 12 control) [14] and GSE104954 (European Renal cDNA Bank tubulointerstitial microarray; 17 diabetic nephropathy and 21 living-donor control) [15]. Single-nucleus RNA sequencing of early human DKD was obtained from GSE131882 (3 control and 3 diabetic kidneys) [16]. Perturbation signatures were drawn from the LINCS L1000 resource [17] as packaged for signatureSearch 1.26.0 [18]. Drug development stage was queried from ChEMBL, trial status from ClinicalTrials.gov, and senescence gene sets (SAUL_SEN_MAYO, FRIDMAN_SENESCENCE_UP/DN) from MSigDB. The three bulk cohorts are distinct GEO series from separate primary studies and platforms (one bulk RNA-seq and two tubulointerstitial microarrays) with no shared samples; complete patient-level independence cannot be confirmed from public metadata. This heterogeneity is deliberate: a senescence-associated signal that persists across whole-kidney RNA-seq and two tubulointerstitial microarray series, measured in different patient populations, is less likely to reflect a platform-specific or cohort-specific artefact than a signal derived from a single series. Three design choices exploit it: per-sample scoring is rank-based and therefore invariant to between-platform differences in dynamic range; the consensus signature requires concordant direction in all three cohorts, so no single platform can impose a gene on the signature; and leave-one-cohort-out analysis quantifies how much of the ranking depends on any one cohort (Section 2.5).

### 2.2. Senescence Quantification and Differential Expression

Because the deposited bulk matrices were log-normalized rather than raw counts, differential expression was performed with limma 3.68.4 using empirical-Bayes moderated statistics with trend and robust options [19]; this choice also matches the established DKD senescence-signature literature [6]. Senescence-associated transcriptional activity was scored per sample by single-sample gene set enrichment analysis (ssGSEA, rank-based normalization) using SenMayo [3] and the Fridman senescence-up set [20]. ssGSEA was computed with GSEApy 1.2.1, and gene sets were retrieved from MSigDB (v2024.1.Hs); set members matched the cohort expression matrices at high rates (SAUL_SEN_MAYO 103/124, 83.1%, in GSE142025; 121/124, 97.6%, in GSE30529; 120/124, 96.8%, in GSE104954; FRIDMAN_SENESCENCE_UP 70/77, 90.9%, 73/77, 94.8%, and 73/77, 94.8%, respectively; Table S3), and microarray probes were mapped to gene symbols, retaining per gene the most variable probe (GSE30529) or the maximal-intensity probe (GSE104954); the RNA-seq matrix contained no duplicate gene symbols and required no collapsing. For the two scored sets, coverage was therefore lowest in the RNA-seq discovery cohort rather than in the microarray cohorts; the 13-gene FRIDMAN_SENESCENCE_DN set, used only for signature construction, had its lowest coverage in GSE104954 (9/13, 69.2%). The deposited GSE142025 matrix excludes transcripts with fewer than 100 reads across all samples, and the absent SenMayo members are predominantly low-abundance secreted SASP factors (among them CCL1, CCL7, CSF2, IL1A, TNF and DKK1); CDKN2A is likewise absent from that matrix. Part of the attenuation of the bulk senescence signal in the discovery cohort is therefore technical rather than biological, and markers that are not detected cannot be interpreted as unexpressed. These procedures were intended to limit platform-driven bias. DKD-versus-control differences were assessed by two-sided Mann–Whitney tests, summarized as rank-biserial effect sizes with 95% bootstrap confidence intervals, and corrected across the six cohort-by-gene-set comparisons with the Benjamini–Hochberg procedure. Whole-transcriptome preranked GSEA was performed for comparison. Each cohort was analyzed independently (within-cohort limma and per-sample rank-based ssGSEA); consequently, no cross-cohort batch correction was applied; rank-based scoring mitigates platform-scale differences.

### 2.3. Signature Construction

Two query signatures were derived from the per-gene limma results, a level of analysis distinct from the per-sample ssGSEA test of Section 2.2, which assesses gene-set-level elevation rather than individual genes. The consensus DKD signature comprised genes with concordant direction across all three cohorts and per-gene significance (adjusted *p* < 0.05) in at least two; the senescence-focused signature was the intersection of these consensus genes with the senescence gene-set universe, retaining the directional split into up- and down-regulated members (the 53 resulting genes are listed in Table S1).

### 2.4. Connectivity Reversal and Prioritization

Each signature was queried against 45,956 LINCS L1000 perturbation signatures with signatureSearch (gess_lincs) [18], and perturbations were ranked by the normalized connectivity score (NCS); compounds with the most negative NCS oppose (reverse) the query. For each query, signatureSearch computes a weighted Kolmogorov–Smirnov enrichment score separately for the up- and down-regulated gene sets against the ranked reference signature (weighting exponent 1); the weighted connectivity score (WTCS) is half the difference between these two scores when they have opposite signs, and zero otherwise; negative values therefore indicate reversal. Significance is obtained empirically against the precomputed null distribution supplied with the package, generated from 1000 random queries of 150 up- and 150 down-regulated genes, and corrected with the Benjamini–Hochberg procedure. NCS normalizes WTCS within cell line, perturbation type and sign, and the cell-summarized score (NCSct) summarizes NCS across cell lines by the maximum-quantile statistic. Default parameters of gess_lincs in signatureSearch 1.26.0 were used, with sortby = NCS and tau = FALSE, so the Tau statistic was not computed. Per-compound reversal was summarized as the mean NCS across cell lines (reported as reversal strength, the mean −NCS, in tables and figures, such that stronger reversal takes positive values), retaining only named compounds with annotated mechanism of action and reversal in at least three cell lines. Candidates were annotated and ordered by reversal strength across both signatures with ChEMBL maximum development phase and ClinicalTrials.gov evidence of prior testing in DKD or chronic kidney disease. The signatureSearch LINCS reference comprises replicate-collapsed Level 5 perturbation signatures; although the reference includes the HA1E kidney-derived epithelial line, it contains no disease-relevant DKD kidney model: the reference comprises immortalized and cancer-derived lines perturbed in vitro, so transcriptome reversal is a similarity heuristic over perturbation space rather than evidence of pharmacological activity in diseased kidney tissue; the mean NCS across available cell lines was therefore used as a cell-type-agnostic summary, and reversal in at least three cell lines was required to avoid single-cell-line artefacts. ClinicalTrials.gov was queried for each prioritized compound (by name and synonyms) together with the terms diabetic kidney disease, diabetic nephropathy, chronic kidney disease and renal fibrosis, and a compound or its mechanistic class was flagged as previously tested when it appeared in a registered DKD or CKD trial.

### 2.5. Validation

As an internal positive control, enrichment of a curated senotherapeutic reference set among signature reversers was tested by preranked GSEA over the full compound ranking. As an external check, reversal scores were tested for their ability to separate 58 curated senolytics from 2465 negative-library compounds assembled independently [9]; discrimination was summarized by the area under the receiver-operating curve (Mann–Whitney U divided by the product of class sizes) and the area under the precision–recall curve, each with a 95% confidence interval from 2000 bootstrap resamples. Prioritization robustness was assessed by recomputing reversal for three leave-one-cohort-out consensus signatures and for an up-regulated-genes-only query (the 1224 up-regulated genes of the consensus signature with an empty down-regulated set), comparing per-compound scores by Spearman correlation and by preservation of the top reversers (Table S2). In an additional sensitivity analysis, cohort-level rank-biserial effects were synthesized separately for each gene set with a random-effects model using bootstrap-derived variances. LINCS candidates were also required, in each query, to have data from at least three cell lines, a negative median cell-summarized connectivity score (NCSct), negative direction in at least two-thirds of cell lines and at least one WTCS signature with FDR < 0.05. HA1E, a kidney-derived epithelial line rather than a DKD model, was examined descriptively where signatures were available.

### 2.6. Exploratory Drug–Target Mendelian Randomization

To add a genetic sensitivity axis independent of the expression-reversal and curated-benchmark analyses, an exploratory drug–target Mendelian randomization was performed for prioritized target genes. Twelve targets were queried: JAK2, TYK2, BIRC5, HDAC1, HDAC2, HDAC3, HDAC6, HDAC8, PTK2, PTK2B, IKBKB and NR3C1. Lead cis-eQTLs for target-gene expression were queried from GTEx v8 [21]. Kidney-cortex eQTL instruments were sought first, but no significant GTEx v8 kidney-cortex cis-eQTLs were returned for the tested targets; therefore, Whole Blood cis-eQTLs were used only as proxy instruments where available. Outcome associations were extracted from the open CKDGen/UK Biobank eGFRcrea GWAS meta-analysis of Stanzick et al. [22]. For each target with an available instrument, alleles were harmonized, and a single-SNP Wald ratio was computed for genetically proxied target expression on eGFR. Because the CKDGen summary statistics report effect estimates and standard errors to four decimal places, each outcome effect was recovered from its reported *p*-value and the sign of the published estimate (|β| = z × SE); *p*-values are therefore unaffected by rounding, whereas effect sizes and confidence intervals are approximate. Instrument strength was summarized by the F-statistic. Because only one lead eQTL was available per estimable target, this analysis was treated as exploratory and could not assess horizontal pleiotropy or colocalization.

### 2.7. Single-Nucleus Analysis

zUMIs dgecounts matrices (intron + exon UMI counts) from the six GSE131882 samples were combined, mapped from Ensembl identifiers to symbols, and processed in Scanpy 1.12.1 [23]. Nuclei were retained at a minimum of 300 detected genes and 500 counts with under 20% mitochondrial content, removing empty droplets; counts were normalized to 10,000 per nucleus and log-transformed; the 2000 most variable genes were selected; principal component analysis used 30 components computed on those genes; the neighbourhood graph used 15 nearest neighbours; Leiden clustering used resolution 0.5 with the igraph backend, two iterations and an undirected graph, which produced the clusters subsequently annotated as nine cell types; and UMAP used the Scanpy defaults (min_dist = 0.5, spread = 1.0). No cross-sample batch integration was applied; instead, the contribution of each sample to every cluster was inspected directly to guard against single-sample artefacts. Clusters were assigned to kidney cell types by marker-set scoring, and senescence was quantified with SenMayo, the Fridman senescence set and individual core markers. Because nuclei from the same donor are not independent observations, condition comparisons were performed using donor-aggregated cell-level scores (not count-summed pseudobulk): for each cell type, per-donor SenMayo and Fridman means, and CDKN1A-positive fractions were compared between 3 control and 3 DKD donors by two-sided Mann–Whitney tests, with nuclei-level distributions shown for description only. To explore, rather than definitively separate, stable arrest from cell activation, endothelium (the cell type carrying the strongest signal) was additionally scored, per donor, for a senescence-arrest panel (CDKN1A, TP53, CDKN2B, GLB1, SERPINE1) and an endothelial-activation panel (ICAM1, VCAM1, VWF, CCL2, CXCL1).

### 2.8. Reproducibility

Analyses were performed in R 4.6.0 (limma 3.68.4, signatureSearch 1.26.0, ExperimentHub 3.2.0) and Python 3.12.13 (GSEApy 1.2.1, Scanpy 1.12.1, anndata 0.12.17, pandas 2.3.3, NumPy 2.4.6, SciPy 1.18.0, scikit-learn 1.9.0, statsmodels 0.14.6). Bootstrap confidence intervals for the rank-biserial effects and for the external benchmark AUROC and AUPRC were computed with the NumPy PCG64 generator seeded with 42 and 2000 resamples; the sensitivity analyses, including the random-effects synthesis and the permutation test of the external benchmark, were seeded with 20260705. Statistical tests, multiple-testing corrections, bootstrap procedures, inferential units and sensitivity analyses are specified in the corresponding Methods subsections. The final versioned analysis archive is available at Zenodo (https://doi.org/10.5281/zenodo.22836503), in accordance with FAIR data principles.

### 2.9. Use of Generative Artificial Intelligence

During the preparation of this study, the authors used OpenAI Codex (GPT-5.6; OpenAI, San Francisco, CA, USA) and Claude (Anthropic, San Francisco, CA, USA; Claude Opus 5) for code review, figure layout preparation and language editing. The tools were not used to generate data, to design the study, or to produce or interpret any analytical result. The authors have reviewed and edited the output and take full responsibility for the content of this publication.

## 3. Results

### 3.1. Senescence-Associated Transcriptional Activity Is Consistently Elevated in DKD at the Per-Sample Level

**Figure 2:**
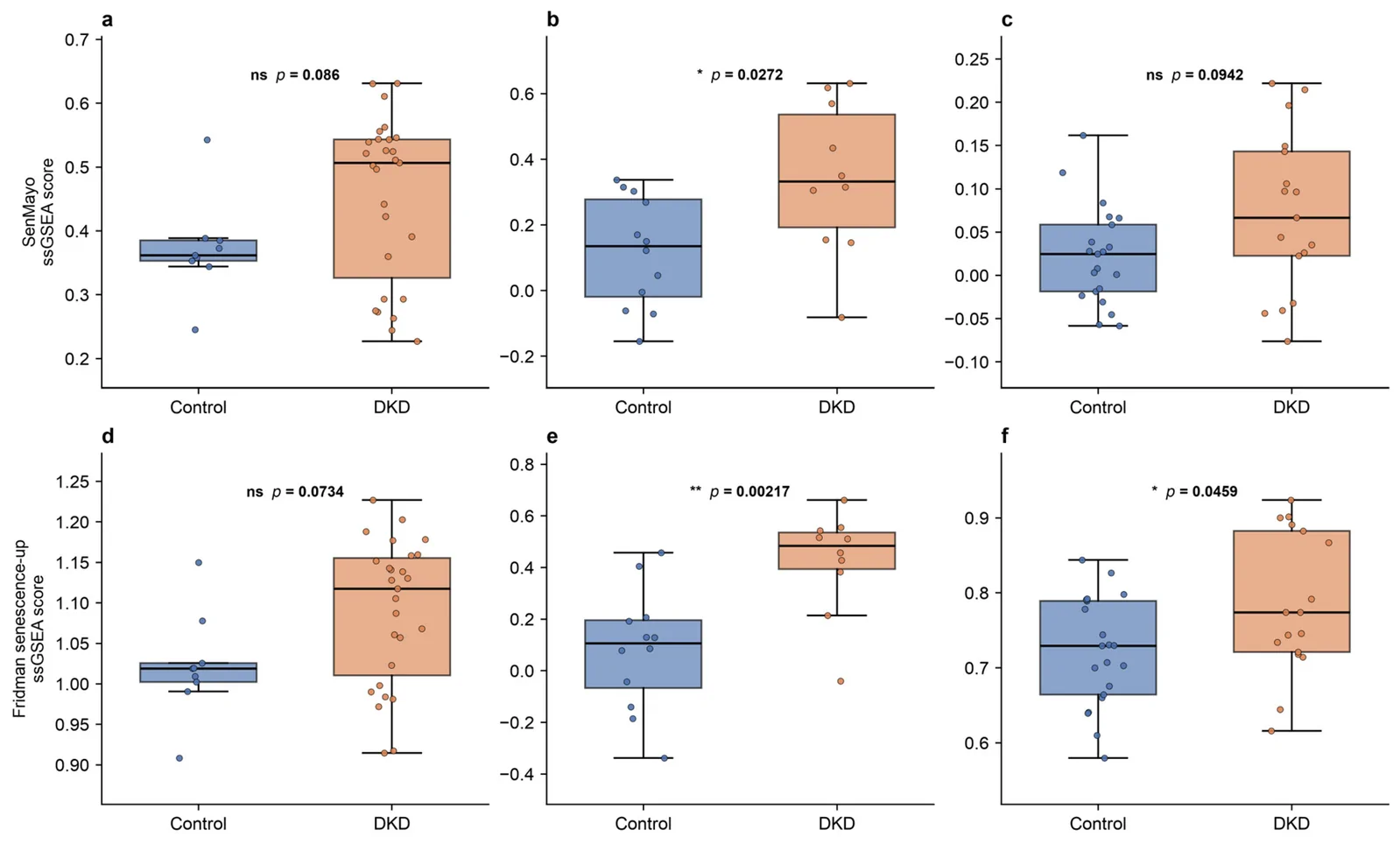
Per-sample senescence-associated transcriptional activity is consistently elevated in DKD. Single-sample gene set enrichment (ssGSEA) scores for the SenMayo (**a**–**c**) and Fridman senescence-up (**d**–**f**) gene sets in control versus DKD kidney across three cohorts: (**a**,**d**) GSE142025 (RNA-seq; 9 control, 27 DKD), (**b**,**e**) GSE30529 (microarray; 12 control, 10 DKD), (**c**,**f**) GSE104954 (microarray; 21 control, 17 DKD). Boxplots show the median and interquartile range with individual samples overlaid (blue, control; orange, DKD). Significance markers are nominal two-sided Mann–Whitney U *p*-values computed on the per-sample ssGSEA scores (ns, not significant; * *p* < 0.05; ** *p* < 0.01); these values are not adjusted for multiple comparisons, and the Benjamini–Hochberg adjusted values across the six cohort-by-gene-set tests are given in Table 2.

The result depended on the enrichment statistic. Whole-transcriptome preranked GSEA did not detect significant senescence enrichment in DKD, which may reflect minority-cell dilution, tissue composition, or differences between enrichment statistics. Per-sample ssGSEA, the approach used in the DKD senescence-signature literature [6], yielded a different and directionally consistent result: senescence-associated transcriptional activity was higher in DKD than control across all three distinct cohorts (Figure 2; Table 2). All six cohort-by-gene-set comparisons were positive in direction, with moderate-to-large effect sizes (rank-biserial 0.32–0.78). After Benjamini–Hochberg correction across the six two-sided tests, all six had BH-adjusted q values below 0.10, although only one met the prespecified conventional 0.05 threshold (GSE30529, Fridman set, FDR = 0.013); the strongest single effect was also in GSE30529 (rank-biserial 0.78, 95% CI 0.45–1.00). An additional random-effects sensitivity synthesis supported this directional consistency for SenMayo (pooled rank-biserial 0.38, 95% CI 0.11–0.60; I^2^ = 0%) and Fridman (0.40, 95% CI 0.11–0.63; I^2^ = 0%). With three heterogeneous cohorts, the heterogeneity estimates are imprecise, and this synthesis is supportive rather than definitive. The pattern is therefore one of consistent, modest elevation rather than a strong effect in any single cohort, and the contrast with the non-significant whole-transcriptome statistic is compatible with, but does not prove, a spatially or cell-type restricted process rather than a tissue-wide transcriptional shift.

Differential expression in the RNA-seq cohort returned 1166 genes (661 up, 505 down at adjusted *p* < 0.05 and absolute log2 fold-change ≥ 1; Figure S1). The most strongly down-regulated genes (FOS, ATF3, the NR4A family and DUSP1) recapitulate immediate-early and stress-response transcripts; FOS is a hub gene of the published DKD senescence signature [6], providing external consistency for the differential-expression layer.

### 3.2. Two Complementary DKD Senescence Signatures

Cross-cohort integration yielded a consensus DKD signature of 1224 up- and 878 down-regulated genes. Restricting these to senescence-annotated genes produced a compact senescence-focused signature of 45 up- and eight down-regulated members, comprising senescence-associated genes that also overlap inflammatory, fibrotic, endothelial and stress programs, including CCL2, CXCL1, MMP1, MMP2, SERPINE2, ICAM1, THBS1, SPP1, IL32 and TP53 (Figure S2; Table S1). The two signatures provide a deliberate trade-off: the senescence-focused signature is more directly anchored to the selected gene sets but is not specific to stable arrest, whereas the consensus signature is broader and more stable, and both were carried forward to avoid dependence of the prioritization on a single definition.

### 3.3. Reversal Recovers Senotherapeutics and Is Modestly Supported by an External Senolytic Benchmark

**Figure 3:**
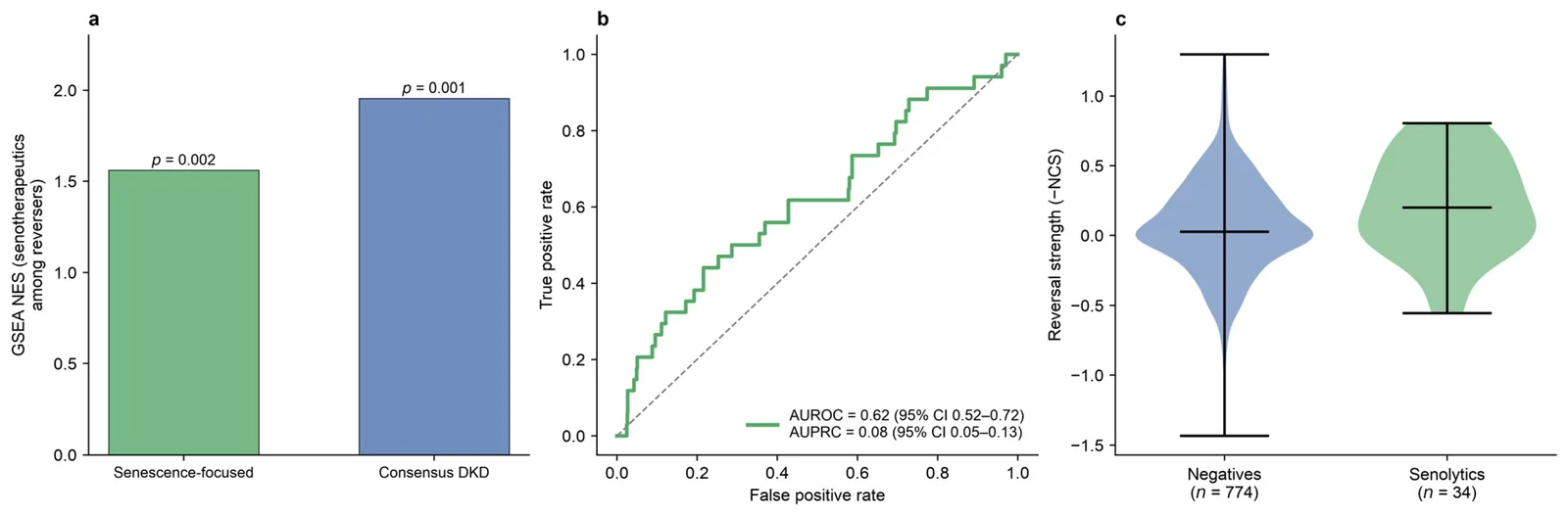
Reversal recovers senotherapeutics and is modestly supported by an external senolytic benchmark. (**a**) Internal positive control: preranked GSEA normalized enrichment score (NES) for a curated senotherapeutic reference set among signature reversers, for the senescence-focused and consensus DKD signatures (nominal *p* shown). (**b**) External benchmark: receiver-operating-characteristic curve separating curated senolytics from a negative library by reversal strength; the area under the curve (AUROC) and the area under the precision–recall curve (AUPRC), each with a 95% bootstrap confidence interval, are shown; the dashed diagonal marks chance performance (AUROC = 0.5). (**c**) Reversal-strength (−NCS) distributions for benchmark negatives (*n* = 774) and senolytics (*n* = 34); horizontal lines mark medians.

Querying the LINCS atlas ranked 45,956 perturbation signatures by connectivity. The first validation tested whether known senotherapeutics were enriched among the reversers, and they were: preranked GSEA over the full ranking returned a normalized enrichment score of 1.56 (*p* = 0.002, false-discovery rate 0.002) for the senescence-focused signature and 1.95 (*p* = 0.001, false-discovery rate 0.001) for the consensus signature (Figure 3a). The control was repeated on the four variants of the consensus signature used for the sensitivity analyses, the three leave-one-cohort-out signatures and the up-regulated-genes-only query, returning normalized enrichment scores of 1.63 to 2.15 (nominal *p* < 0.03) against 1.95 for the full consensus signature, so the enrichment does not depend on any single cohort or on the down-regulated half of the query. One methodological point warrants emphasis. A query of a top-50 connectivity service, whose output has limited ranking resolution, did not reach significance against random signatures (bootstrap *p* = 0.37 and 0.94), whereas full-ranking statistics detected the enrichment. The difference reflects the granularity of the statistic rather than biology and is relevant to the reproduction of this class of analysis.

The second validation was performed outside the present data. Reversal scores were tested against an independently curated benchmark of 58 senolytics and 2465 negative-library compounds [9]. Among the 808 compounds shared with the LINCS ranking (34 senolytics, 774 negatives), reversal strength separated the two classes with an area under the curve of 0.62 (95% CI 0.52–0.72) and an area under the precision–recall curve of 0.08 (95% CI 0.05–0.13; positive-class prevalence 0.04; Figure 3b,c). The separation was unlikely to arise under random labelling (label-permutation *p* = 0.009, 10,000 permutations). The discrimination is modest, with a lower confidence bound close to chance, as expected for a transcriptome-only predictor. Two considerations limit the independence of this evidence. First, the benchmark positives, like the internal reference set, are the literature-curated senotherapeutics; the two validations therefore address a correlated question rather than two orthogonal ones. Second, an experimental phenotypic senolytic screen [10] could not be accessed for an orthogonal test. The compounds driving the separation, namely cardiac glycosides (bufalin, ouabain, digitoxin, digoxin, proscillaridin), panobinostat, HSP90 inhibitors and dasatinib, are nonetheless recognized senotherapeutics, several validated experimentally [24], indicating that the ranking reflects recognized senotherapeutic activity.

### 3.4. A Prioritized, Druggability- and Novelty-Annotated Candidate Map

**Figure 4:**
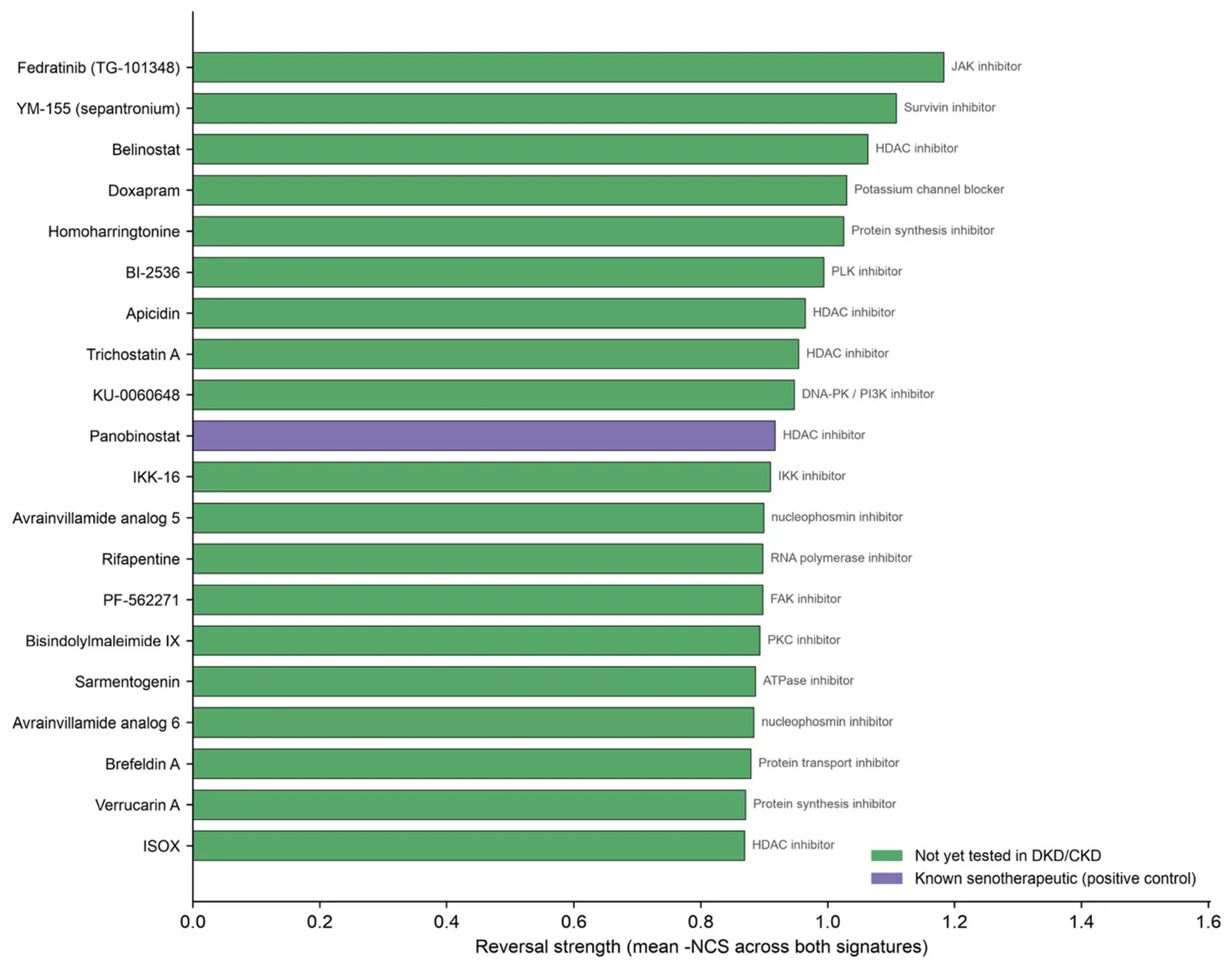
Transcriptome-reversal candidate map. The 20 strongest compounds reversing the DKD senescence-associated signatures, ranked by reversal strength (mean −NCS across the senescence-focused and consensus signatures) and annotated by mechanism of action. Bar colour denotes the registry-search annotation: green, no DKD/CKD trial identified in the prespecified search; purple, an established senotherapeutic included as an internal positive control. The ranking reflects computational reversal strength and is not a measure of potency, efficacy or suitability for clinical use.

Combining the two signatures yielded 867 named, mechanism-annotated compounds that reverse both DKD senescence-associated transcriptional signatures in both analyses (Figure 4; Table 3). The ten compounds listed in Table 3 span inhibitors of Janus kinase (JAK), histone deacetylase (HDAC), polo-like kinase (PLK), focal adhesion kinase (FAK), survivin/BIRC5 and IKK/NF-κB, together with a glucocorticoid receptor agonist. This filtered candidate universe reflects sequential filtering: of 8140 LINCS perturbagens profiled across 30 cell lines (45,956 signatures), 3860 were named (non-BRD), and 867 of these reversed both the senescence-focused and consensus signatures in at least three cell lines with an annotated mechanism of action. Overall reversal scores were stable to these analytical choices, although upper-tail membership varied: per-compound reversal scores from three leave-one-cohort-out signatures and from an up-regulated-genes-only query correlated strongly with the full analysis (Spearman ρ = 0.81–0.87); all 50 top reversers remained reversers, and all ten compounds in Table 3 were preserved (Table S2). An additional, stricter sensitivity filter retained 160 of the 867 candidates. Eight of the ten compounds in Table 3 passed this filter; PF-562271 and methylprednisolone did not, because neither query contained an FDR-supported consensus signature for those compounds. HA1E signatures were available for four of the ten compounds, precluding a general kidney-line validation; among those four, support was compound- and query-dependent. The stricter filter therefore provides ranking sensitivity rather than disease- or cell-type-specific validation. Several observations follow from this list.

First, the internal controls performed as intended: cardiac glycosides, geldanamycin, everolimus and dasatinib ranked highly, the same classes that accounted for the separation in the external benchmark. Their recovery supports the calibration of the ranking but is not novel.

Second, among approved drugs, histone deacetylase (HDAC) inhibitors recurred more than any other class: belinostat, panobinostat, romidepsin and vorinostat are all approved (ChEMBL maximum phase 4), and additional HDAC inhibitors (apicidin, trichostatin A, the HDAC6-selective compound ISOX) appeared independently. The convergence of multiple chemically distinct HDAC inhibitors on the same signature is internally robust. This is not, however, a novel proposition for the kidney: a PubMed search returns 145 records for HDAC or histone deacetylase combined with diabetic kidney disease, diabetic nephropathy or renal fibrosis (as of June 2026); the appropriate claim is therefore that an unbiased senescence-reversal analysis independently prioritizes a class already under investigation, while adding that, within the scope of the ClinicalTrials.gov search described in Section 2.4, no HDAC inhibitor has yet been tested in a DKD or chronic kidney disease clinical trial.

Third, the JAK inhibitor fedratinib was the single strongest reverser among approved drugs. This recovery has pathway-level clinical proof-of-concept, but not compound-level validation: baricitinib, a JAK1/2 inhibitor, reduced albuminuria by 41% in a Phase 2 DKD trial [4]. The result supports the disease relevance of the JAK pathway; it does not establish fedratinib as clinically suitable for chronic DKD because its selectivity, exposure and safety profile differ from baricitinib.

Fourth, the analysis nominated mechanisms with little or no prior DKD testing, which are the principal candidates for further study: survivin/BIRC5 inhibition (YM-155, Phase 2; a high-risk experimental hypothesis with no kidney-disease trials identified), FAK inhibition (PF-562271), IKK/NF-κB inhibition (IKK-16, consistent with SASP suppression) and glucocorticoid receptor agonism (methylprednisolone). Cross-referencing ClinicalTrials.gov confirmed that the senotherapeutics already trialled in chronic kidney disease (dasatinib plus quercetin, NCT02848131; fisetin, NCT03325322) are recovered but fall outside the novel set, supporting the calibration of the prioritization framework. Candidates also span a tractability gradient that the ranking does not by itself encode: mechanistically plausible pathways that still require compound-level benefit–risk assessment (the JAK axis), mechanistically relevant but toxicity-limited classes (HDAC and survivin/BIRC5 inhibitors), and probable nonspecific or cytotoxic perturbagens (for example homoharringtonine, brefeldin A and verrucarin A) that likely reverse a generic stress or protein-synthesis signature and should be down-weighted for chronic-disease repurposing.

### 3.5. Exploratory Drug–Target Mendelian Randomization Is Instrument-Limited and Inconclusive

**Figure 5:**
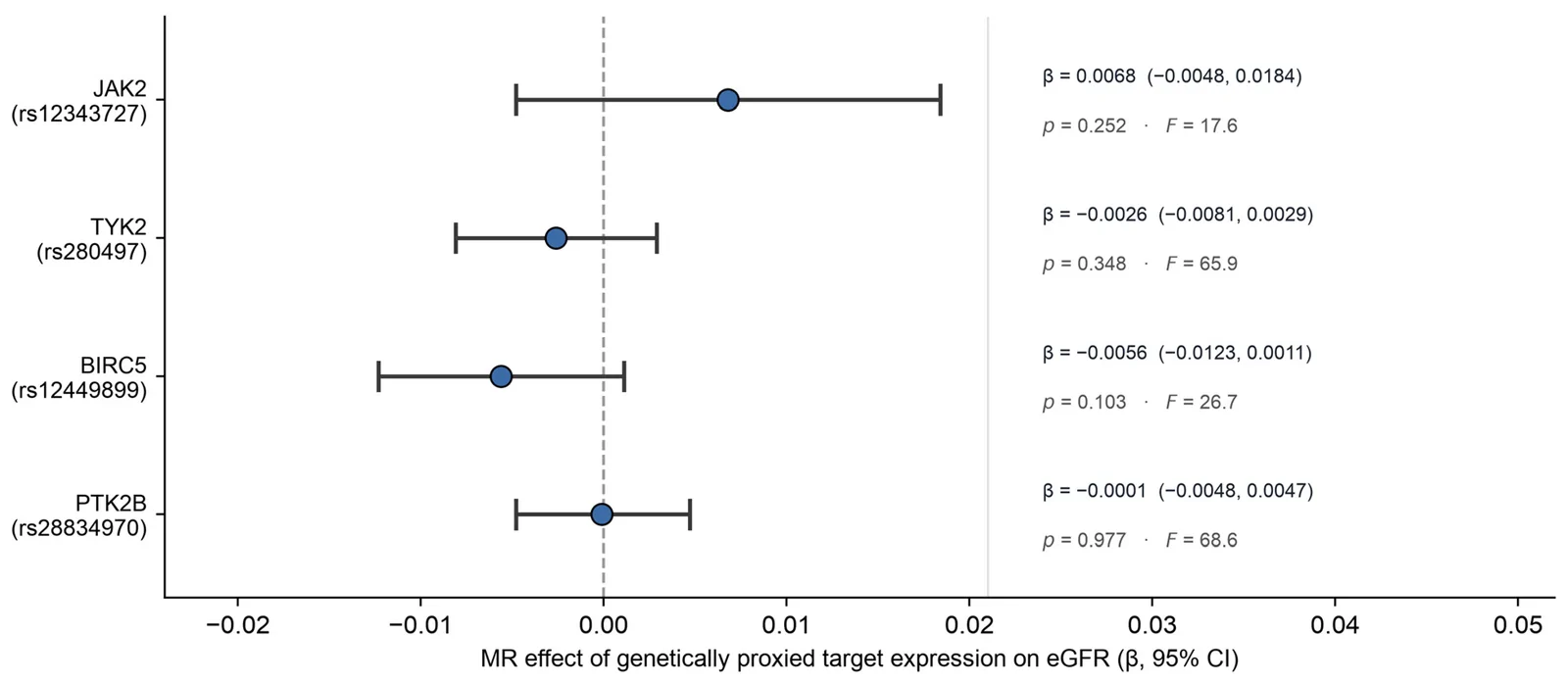
Exploratory drug–target Mendelian randomization. Single-SNP Wald-ratio estimates (β with 95% CI) of genetically proxied target-gene expression on eGFR for the four targets with an available instrument (GTEx v8 Whole Blood cis-eQTL; lead variant per target). Instrument strength is given as the F-statistic; the dashed line marks the null. No tested target reached statistical significance.

The genetic sensitivity analysis did not support any of the tested target associations. Among 12 prioritized target genes queried, no significant GTEx v8 kidney-cortex cis-eQTL instrument was available. Whole Blood proxy instruments were available for four targets: JAK2, TYK2, BIRC5 and PTK2B (F-statistics 17.6–68.6; Figure 5 and Table 4). None showed a significant Wald-ratio association with eGFR after allele harmonization. The directions for TYK2 and BIRC5 were compatible with lower expression being associated with higher eGFR, but the confidence intervals crossed the null (BIRC5, β = −0.0056, 95% CI −0.0123 to 0.0011, *p* = 0.103; TYK2, β = −0.0026, 95% CI −0.0081 to 0.0029, *p* = 0.348). JAK2 showed the opposite direction for pharmacologic inhibition and was also non-significant (β = 0.0068, 95% CI −0.0048 to 0.0184, *p* = 0.252). Thus, drug–target Mendelian randomization does not materially strengthen the candidate claims; rather, it provides a transparent negative sensitivity layer and highlights the need for kidney-relevant QTL or protein-QTL instruments before genetic causal support can be asserted.

### 3.6. Single-Nucleus Context: A Donor-Consistent Senescent Compartment Was Not Resolved

**Figure 6:**
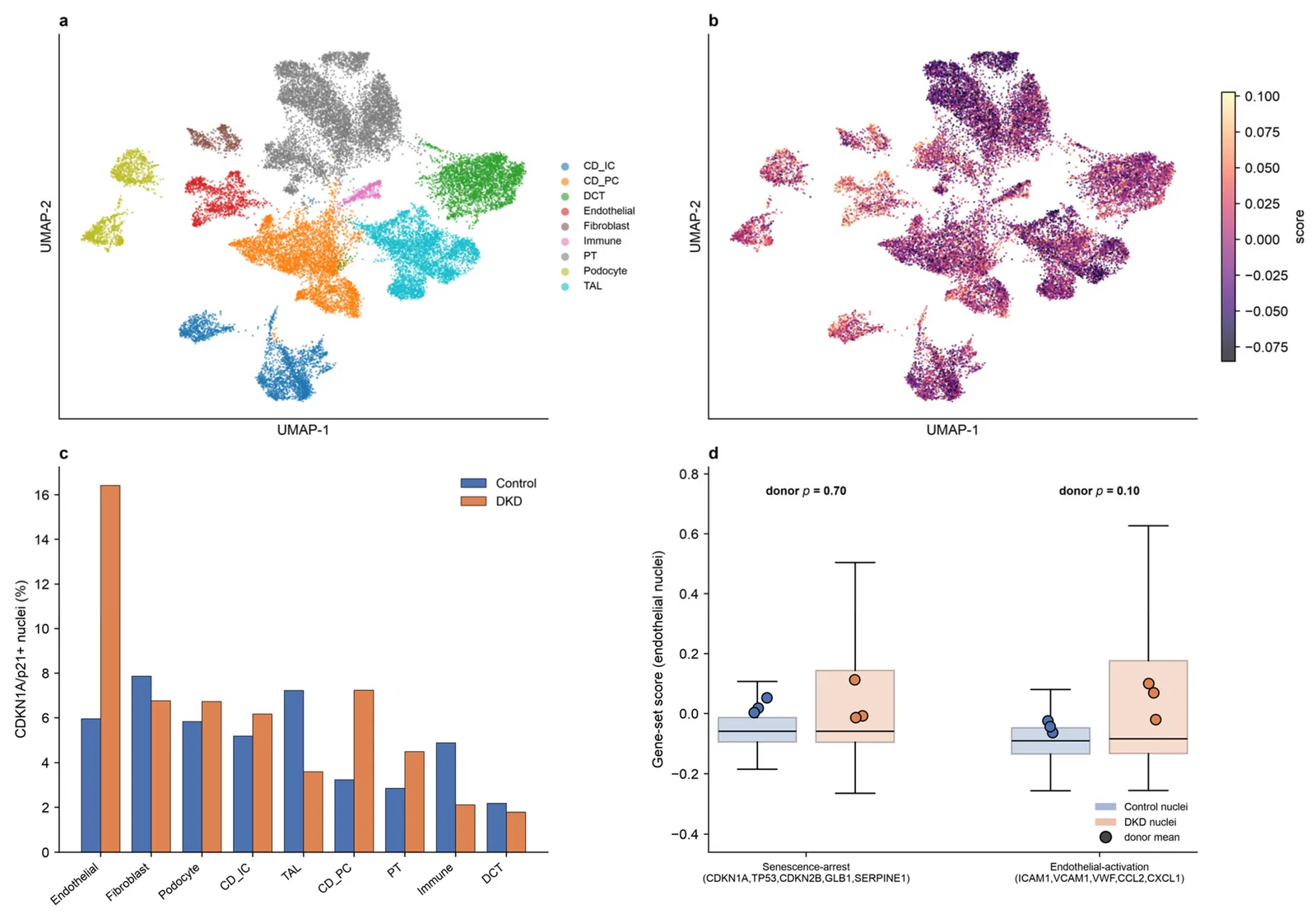
Single-nucleus context of the DKD senescence-associated signal (GSE131882). (**a**) Cell-type UMAP of 25,104 nuclei resolved into nine kidney cell types. (**b**) SenMayo senescence-associated score per nucleus on the same embedding. (**c**) Descriptive percentage of CDKN1A/p21-positive nuclei by cell type (blue, control; orange, DKD). (**d**) Donor-level endothelial gene-set scores for a senescence-arrest panel (CDKN1A, TP53, CDKN2B, GLB1, SERPINE1) and an endothelial-activation panel (ICAM1, VCAM1, VWF, CCL2, CXCL1): light boxes show nuclei distributions (descriptive) and filled points show the three control and three DKD donor means (the inferential units). To avoid pseudoreplication, conditions were compared at the donor level (*n* = 3 versus 3); two-sided Mann–Whitney *p*-values are shown.

The bulk signal raised the question of localization, which single-nucleus profiling was used to address. After quality control, 25,104 nuclei (14,482 control, 10,622 DKD) resolved into nine kidney cell types, with proximal tubule the most abundant (7184 nuclei); endothelial nuclei were distributed across all six donors (110–324 per donor). Because nuclei from the same donor are not independent, comparisons were made at the donor level (three control versus three DKD). At this level, the analysis could not resolve a donor-consistent senescent compartment: neither proximal tubule (SenMayo, donor-level *p* = 0.70) nor endothelium (SenMayo, *p* = 1.0) showed a senescence-score difference, with similarly non-significant donor-level results for the Fridman senescence set. CDKN1A/p21-positive endothelial nuclei were numerically higher in DKD, whether nuclei were pooled irrespective of donor (5.95% of control versus 16.43% of DKD nuclei) or averaged across donors (8.41% versus 16.38%; the two DKD estimates differ by 0.05 percentage points and both round to 16.4%) but this was not supported at the donor level (*p* = 1.0) and was driven by a single DKD donor, and it is therefore reported descriptively. An exploratory panel comparison provided additional context: across donors an endothelial-activation panel (ICAM1, VCAM1, VWF, CCL2, CXCL1) was higher in all three DKD donors than in all three controls (complete separation; donor-level *p* = 0.10, the minimum attainable at *n* = 3 versus 3), whereas a senescence-arrest panel (CDKN1A, TP53, CDKN2B, GLB1, SERPINE1) did not differ (*p* = 0.70; Figure 6), and the canonical drivers CDKN2A/p16 and IL6 were undetected. The endothelial pattern may therefore reflect activation rather than stable senescence arrest, although with three donors per group this analysis is exploratory. The two states cannot be separated definitively with these data: CDKN1A/p21 is induced by stress and by endothelial activation as well as by senescence and is therefore not discriminative on its own, and CDKN2A/p16 and IL6 were undetected. The claim is accordingly bounded: endothelial senescence is not excluded, but the only cross-donor-consistent endothelial signal in this dataset is an activation signature.

Two cautious conclusions can be drawn. First, at single-nucleus resolution in this early-DKD cohort, the tissue-level senescence signal could not be localized to a donor-consistent senescent population; the most consistent cross-donor cell-type difference, in endothelial cells, may reflect activation rather than arrest, and p21 induction alone, being stress- and activation-inducible, does not establish senescence. Second, this raises the possibility that the bulk reversal signature blends compartments and partly captures inflammatory programs, and that compartment-resolved profiling with arrest-specific and experimentally anchored senescence markers is required before cell-type-targeted senotherapeutic claims can be made in DKD.

## 4. Discussion

This study makes three contributions to senotherapeutic repurposing for DKD: a disease-anchored, multi-cohort prioritization framework; an externally checked, development-stage and novelty-annotated candidate map; and a cautious single-nucleus result that qualifies a common assumption about where the DKD senescence signal resides. The evidence base for each is stated explicitly. The underlying senescence program is directionally consistent across all three cohorts and formally significant after correction in one; its magnitude is modest, as expected for a minority-cell phenomenon. Reversing it against the LINCS atlas recovers established senotherapeutics and ranks a structured shortlist by mechanism, approval status and trial novelty. At single-nucleus resolution, the tissue-level signal did not resolve a donor-consistent senescent compartment; the most consistent cross-donor cell-type difference, in endothelial cells, may reflect activation rather than a senescence-arrest program, a finding that constrains cell-type-specific interpretation and identifies a confound relevant to the wider subfield. That confound is not specific to the diabetic kidney: wherever chronic metabolic stress and advancing age act on the same vascular bed, activation and arrest programs are engaged together, and compact marker panels may therefore attribute to senescence a signal that belongs to endothelial activation, or the reverse.

The contribution is deliberately framed against its predecessors. Luo et al. [6] established a DKD senescence signature but did not extend it to therapeutics; the present work takes that step. Meiners et al. [7] and Olascoaga et al. [8] applied transcriptome-reversal and network methods to senescence in general, but not to a specific disease context with cross-cohort consensus and external benchmarking. The value added here is integrative rather than methodological: a disease-anchored, multi-cohort, externally benchmarked prioritization with explicit druggability and novelty annotation, plus single-cell localization.

The limits of the present claims are equally important. The most prominent recovered classes, HDAC inhibitors and JAK inhibition, are already under investigation in kidney disease, with the JAK pathway supported by human albuminuria data [4]. Presenting them as discoveries would misrepresent the field; they are better understood as convergent confirmation that the pipeline recovers established disease biology, which supports the credibility of its under-explored nominations. The recovery of JAK inhibition is consistent with pathway-level human evidence from baricitinib, but this evidence should not be transferred to fedratinib without compound-specific pharmacology and safety assessment.

Three features of the design bound what the candidate map can mean. First, the senescence-focused signature is built from genes that also belong to inflammatory, fibrotic, endothelial-activation and stress programs; this is a property of transcriptome-derived senescence sets rather than of this particular selection, and it is why prioritization required reversal of both the senescence-focused and the broader consensus signature. A compound that reverses them may therefore act on senescence, on the overlapping programs, or on both. Second, transcriptome reversal scores transcriptional opposition rather than cell fate, so it cannot distinguish a senolytic from a senomorphic mechanism; separating the two requires viability and SASP read-outs in senescent cells. Third, the LINCS reference comprises immortalized and cancer-derived lines perturbed in vitro and contains no diabetic kidney model, so connectivity is a similarity heuristic over perturbation space rather than evidence of activity in diseased kidney tissue. These constraints are the reason the output is presented as a prioritized set of testable hypotheses.

Several limitations constrain the conclusions. The senescence effect in bulk tissue is modest: after multiple-testing correction it is formally significant in one of three cohorts while remaining directionally consistent in all three, and it is detectable only with per-sample scoring, consistent with either a minority-cell process or overlap with tissue-composition and inflammatory programs. Donor age is not deposited for the bulk cohorts, so the senescence scores could not be adjusted for chronological age at the individual level. Where the source publications report age at group level, the direction of the difference is not consistent across cohorts: in GSE30529 the diabetic group was on average 9.4 years older than the control group (63.5 versus 54.1 years, *p* = 0.15), which would tend to raise senescence-associated activity in that cohort, whereas in GSE142025 the control group was the older one (60.9 versus 50.7 and 53.2 years, *p* = 0.63), which would tend to reduce the observed difference. An age contribution to the cohort that reaches the conventional significance threshold therefore cannot be excluded. The elevation is nevertheless in the same direction in GSE142025, where the control group is the older one, so the cross-cohort pattern is not explained by age. The deposited discovery-cohort matrix further lacks CDKN2A and a set of low-abundance secreted SASP transcripts, so part of the attenuation of the bulk signal in that cohort is technical rather than biological. The bulk cohorts also differ in tissue compartment (one whole-kidney and two tubulointerstitial) and array or sequencing platform, which may limit cross-cohort comparability. The two validations both rely on the literature-curated senotherapeutic labels and are therefore not fully independent; the external discrimination is modest (area under the curve 0.62, 95% CI 0.52–0.72, lower bound near chance), and an orthogonal experimental phenotypic screen [10] could not be accessed. The exploratory Mendelian-randomization layer was also limited: GTEx kidney-cortex instruments were unavailable, Whole Blood proxy instruments existed for four of the twelve targets, and single-SNP Wald ratios cannot distinguish causality from linkage or pleiotropy without colocalization. The pipeline is entirely computational, with no wet-laboratory validation, and several top-ranked compounds (for example, panobinostat) carry toxicity profiles that complicate use in a chronic disease. The single-nucleus analysis derives from a single early-DKD dataset with only three donors per group; condition comparisons were therefore made at the donor level and remain exploratory; it lacks p16 and IL6 detection, and suggests that the strongest cell-type signal may overlap endothelial activation; cell-type-resolved senescence claims therefore await arrest-specific markers, additional cohorts and advanced-stage tissue, and the compact arrest and activation panels used to separate the two programs were partly overlapping and would be strengthened by established reference signatures. Because single-nucleus data are sparse, undetected markers such as p16 and IL6 cannot be read as biological absence, and inferences from small gene panels remain tentative. Finally, the stricter NCSct, directional-consistency and FDR sensitivity filter did not remove broadly cytotoxic or stress-inducing perturbagens, showing that connectivity calibration alone cannot establish senescence specificity. Transcriptome reversal cannot by itself separate senomorphic from senolytic activity; the candidate universe should therefore be read as hypothesis prioritization rather than evidence of senolysis.

The practical output is a ranked, annotated candidate map that can guide hypothesis-driven follow-up rather than a single claimed drug. The most informative next steps are compartment-resolved reversal signatures built from senescent endothelial cells, kidney-relevant QTL or protein-QTL colocalization for nominated targets, and targeted experimental testing of the under-explored, lower-toxicity nominations in senescent-cell models.

## 5. Conclusions

Starting from open transcriptomic data alone, a senescence-associated transcriptome-reversal pipeline yields an externally benchmarked, development-stage-annotated universe of candidate senotherapeutic mechanisms and compounds for DKD, together with a cautious single-cell result: the tissue-level senescence signal did not resolve a donor-consistent senescent compartment and showed an exploratory endothelial-activation signal. Within this framework, two leads are selected for follow-up. JAK inhibition has the strongest pathway-level convergence (recovered computationally and supported by Phase 2 albuminuria data for baricitinib [4]), while fedratinib remains a compound-level computational nomination with an unfavourable chronic-DKD benefit–risk profile; among the under-explored nominations, survivin (BIRC5) inhibition is a high-risk experimental hypothesis that requires target-engagement and senescent-cell-selectivity testing in senescent kidney-cell models, with the caveat that current tool compounds carry their own developmental and toxicity limitations. The mechanistic classes recovered are largely those with prior experimental or clinical support; the work should therefore be understood not as the discovery of new drug classes but as a calibrated computational framework that confirms disease-relevant biology and points to a small set of testable, prioritized options.

## Figures and Tables

**Table 1:** Cohorts analyzed. Group sample sizes are shown for each cohort. Age is the group mean in years and sex is the male:female ratio, given as control/DKD; nr, not reported. Age and sex are not deposited in GEO for the three bulk cohorts: the values for GSE142025 and GSE30529 are the published cohort statistics [13,14], with those for GSE142025 referring to the published groups (control, early and advanced diabetic nephropathy), of which the 27 DKD profiles analyzed here are the deposited subset. Individual-level clinical data for GSE104954 are not publicly available. Values for GSE131882 are taken from the deposited per-sample metadata. Controls in GSE104954 were living-donor biopsies. ^a^: Published cohort of 28 DKD patients (6 early and 22 advanced diabetic nephropathy), of which 27 deposited profiles were analyzed.

Accession	Platform	Tissue	DKD	Control	Age (Years), Control/DKD	Sex M:F, Control/DKD
GSE142025	Bulk RNA-seq	Whole kidney	27	9	60.9/50.7 (early), 53.2 (advanced) ^a^	7:2/18:10 ^a^
GSE30529	Affymetrix microarray	Tubulointerstitium	10	12	54.1/63.5	6:6/2:8
GSE104954	Affymetrix microarray (ERCB)	Tubulointerstitium	17	21	nr	nr
GSE131882	Single-nucleus RNA-seq	Kidney (nuclei)	3	3	59.0/61.0	2:1/2:1

**Table 2:** Cross-cohort senescence statistics (per-sample ssGSEA, DKD versus control). Effect = rank-biserial correlation (95% bootstrap CI); *p* = two-sided Mann–Whitney; FDR = Benjamini–Hochberg across the six tests.

Cohort	Gene Set	DKD Median	Control Median	Effect (95% CI)	*p*	FDR
GSE142025	SenMayo	0.507	0.362	0.39 (0.01–0.73)	0.086	0.094
GSE142025	Fridman-up	1.118	1.019	0.41 (0.04–0.74)	0.073	0.094
GSE30529	SenMayo	0.333	0.136	0.57 (0.13–0.90)	0.027	0.082
GSE30529	Fridman-up	0.484	0.107	0.78 (0.45–1.00)	0.002	0.013
GSE104954	SenMayo	0.067	0.025	0.32 (−0.06–0.66)	0.094	0.094
GSE104954	Fridman-up	0.774	0.729	0.38 (0.01–0.71)	0.046	0.092

**Table 3:** Top prioritized compounds reversing the DKD senescence-associated signatures. Reversal is the mean −NCS across the two queries; the number of LINCS cell lines contributing to each compound is shown. ChEMBL maximum phase is a development-stage annotation, not evidence of suitability for chronic DKD. Key targets are annotated from ChEMBL/LINCS, and clinical selectivity may be narrower.

Compound	Mechanism	Key Targets	Cell Lines	Reversal Strength (Mean −NCS)	ChEMBL Max Phase (Status)	Status
Fedratinib (TG-101348)	JAK inhibitor	JAK2, FLT3	12	1.18	4 (approved)	Class clinically supported (baricitinib)
YM-155 (sepantronium)	Survivin/BIRC5 inhibitor	BIRC5	6	1.11	2 (investigational)	Not tested in DKD/CKD
Belinostat	HDAC inhibitor	HDAC1-11	8	1.06	4 (approved)	HDAC class; not tested in DKD
BI-2536	PLK inhibitor	PLK1-3	13	0.99	2 (investigational)	Not tested in DKD
Apicidin	HDAC inhibitor	HDAC1-11	14	0.96	Tool (preclinical)	HDAC class
Trichostatin A	HDAC inhibitor	HDAC1-10	23	0.95	1 (investigational)	HDAC class
Panobinostat	HDAC inhibitor	HDAC1-11	15	0.92	4 (approved)	Known senotherapeutic (control)
IKK-16	IKK/NF-κB inhibitor	IKBKB	8	0.91	Tool (preclinical)	Not tested in DKD (SASP-consistent)
PF-562271	FAK inhibitor	PTK2/PTK2B	12	0.90	1 (investigational)	Not tested in DKD
Methylprednisolone	Glucocorticoid receptor agonist	NR3C1	4	0.86	4 (approved)	Senomorphic

**Table 4:** Exploratory cis-eQTL drug–target Mendelian randomization against eGFRcrea for four targets, using one lead cis-eQTL per target. Exposure is the GTEx v8 Whole Blood target-gene expression proxy; outcome is CKDGen/UKB eGFRcrea. Estimates are single-SNP Wald ratios with 95% confidence intervals; no tested target reached statistical significance.

Target	Proxy Tissue	Lead Cis-eQTL	F-Statistic	MR β Expression-to-eGFR (95% CI)	*p*	Directional Interpretation (Exploratory; All Non-Significant)
JAK2	Whole Blood	rs12343727	17.6	0.0068 (−0.0048 to 0.0184)	0.252	Direction nominally opposes inhibition; not significant
TYK2	Whole Blood	rs280497	65.9	−0.0026 (−0.0081 to 0.0029)	0.348	Nominally compatible with lower expression and higher eGFR; not significant
BIRC5	Whole Blood	rs12449899	26.7	−0.0056 (−0.0123 to 0.0011)	0.103	Nominally compatible with lower expression and higher eGFR; not significant
PTK2B	Whole Blood	rs28834970	68.6	−0.0001 (−0.0048 to 0.0047)	0.977	No directional effect; not significant

## Data Availability

The final versioned analysis archive is available at Zenodo (https://doi.org/10.5281/zenodo.22836503). All primary data are publicly available: Gene Expression Omnibus accessions GSE142025, GSE30529, GSE104954 and GSE131882; the LINCS L1000 resource; MSigDB gene sets; ChEMBL; ClinicalTrials.gov; GTEx v8; the CKDGen/UK Biobank eGFR meta-analysis; and the curated senolytic benchmark (https://doi.org/10.5281/zenodo.7870357). No new data were created in this study.

## Supplementary Materials

The following supporting information can be downloaded at: https://www.mdpi.com/article/10.3390/biomedicines14102286/s1. Figure S1: Differential expression in the diabetic kidney (GSE142025); Figure S2: Senescence-focused signature across cohorts; Table S1: Senescence-focused DKD signature (53 genes); Table S2: Sensitivity of the prioritization to leaving out each cohort and to an up-regulated-genes-only query; Table S3: Senescence gene-set coverage per cohort.

## Abbreviations

The following abbreviations are used in this manuscript:

Abbreviation	Definition
AUPRC	Area under the precision–recall curve
AUROC	Area under the receiver-operating-characteristic curve
CKD	Chronic kidney disease
DKD	Diabetic kidney disease
eGFR	Estimated glomerular filtration rate
eQTL	Expression quantitative trait locus
FAK	Focal adhesion kinase
FDR	False discovery rate
GSEA	Gene set enrichment analysis
HDAC	Histone deacetylase
IKK	IκB kinase
JAK	Janus kinase
LINCS	Library of Integrated Network-Based Cellular Signatures
MR	Mendelian randomization
NCS	Normalized connectivity score
NCSct	Cell-summarized normalized connectivity score
NES	Normalized enrichment score
NF-κB	Nuclear factor kappa B
PLK	Polo-like kinase
SASP	Senescence-associated secretory phenotype
ssGSEA	Single-sample gene set enrichment analysis
UMAP	Uniform manifold approximation and projection
WTCS	Weighted connectivity score
