# Supplementary material for: Senescence-Associated Transcriptome Reversal Prioritizes Repurposing Candidates for Diabetic Kidney Disease: A Computational Study
Source: Biomedicines. 2026 Oct 9;14(10):2286. doi: 10.3390/biomedicines14102286 (PMC13701380; doi:10.3390/biomedicines14102286)
Supplement: Supplementary file 1 [file biomedicines-14-02286-s001.zip › biomedicines-4605336-supplementary.pdf]

Supplementary Materials

Senescence-Associated Transcriptome Reversal Prioritizes Repurposing Candidates for Diabetic Kidney Disease: A Computational Study

Duygu Aygüneş-Jafari [1,*] and Zekeriya Düzgün [2]

[1] Department of Medical Biology, Faculty of Medicine, Ege University, 35100 Izmir, Turkey; duygu.aygunes@ege.edu.tr

[2] Department of Medical Biology, Faculty of Medicine, Giresun University, 28200 Giresun, Turkey; zekeriya.duzgun@giresun.edu.tr

* Correspondence: duygu.aygunes@ege.edu.tr

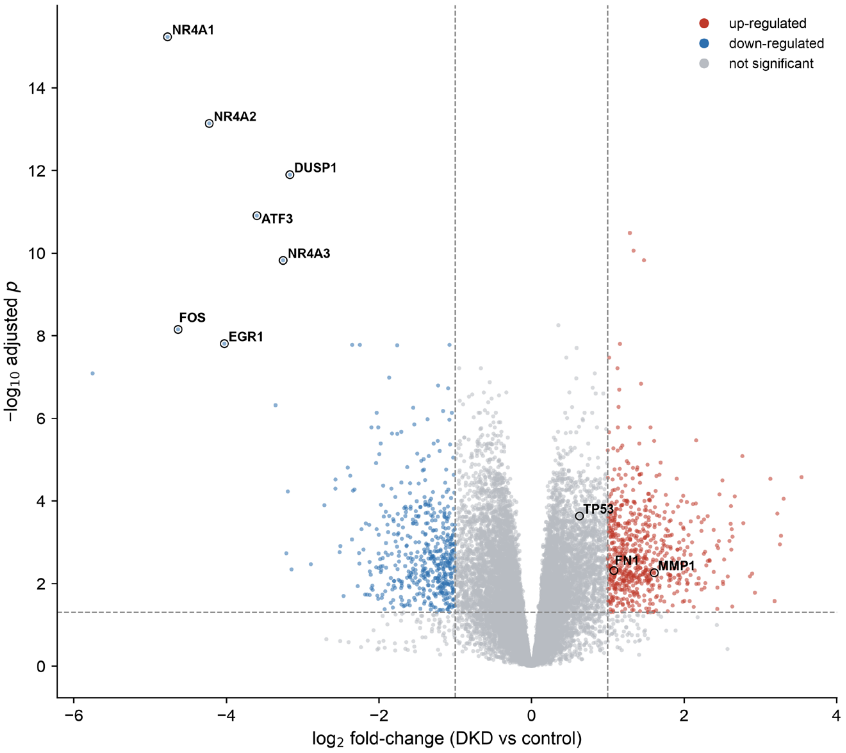

Figure S1. Differential expression in the diabetic kidney (GSE142025). Volcano plot of limma moderated log2 fold-changes (DKD versus control) against −log10 adjusted *p*. Colour key: red, up-regulated; blue, down-regulated (adjusted $p < 0.05$ and $|\log2 \text{ fold-change}| \geq 1$; 661 up, 505 down); grey, not significant. Dashed lines mark the significance thresholds. Labelled genes include the strongly down-regulated immediate-early/stress transcripts (FOS, ATF3, NR4A1-3, DUSP1, EGR1) and selected up-regulated senescence-signature genes (FN1 and MMP1; TP53 is up-regulated but below the fold-change threshold).

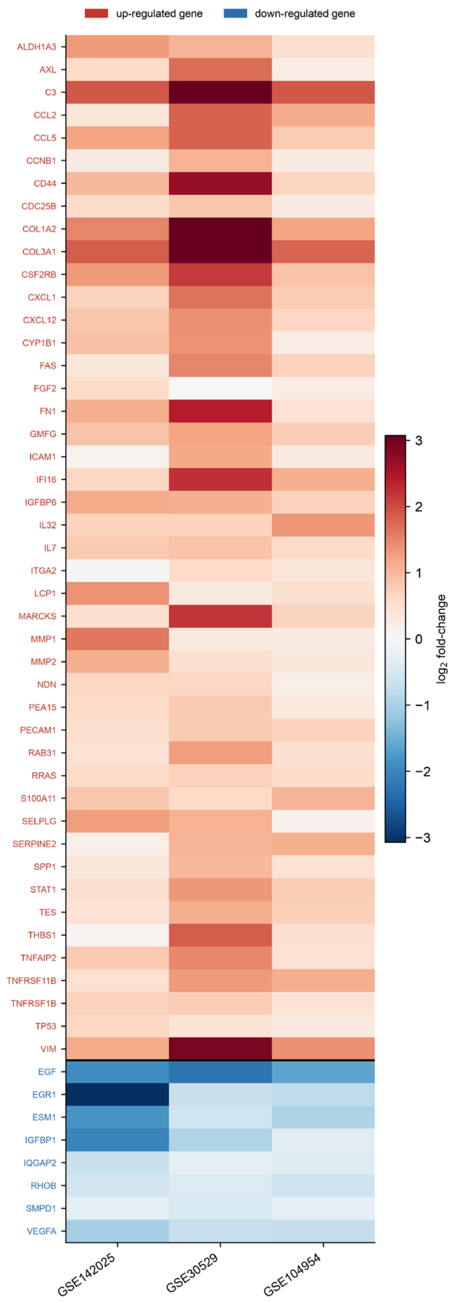

Figure S2. Senescence-focused signature across cohorts. Heatmap of limma log2 fold-changes (DKD versus control) for the 53 senescence-focused signature genes across the three transcriptomic cohorts (GSE142025, GSE30529, GSE104954). Gene-label colour key: red, up-regulated members ($n = 45$); blue, down-regulated members ($n = 8$); the horizontal line marks the up/down divide. The colour bar encodes the log2 fold-change; the direction is concordant across cohorts.

Table S1. Senescence-focused DKD signature (53 genes): direction and per-cohort limma log2 fold-change (GSE142025, GSE30529, GSE104954).

Gene	Direction	logFC GSE142025	logFC GSE30529	logFC GSE104954
ALDH1A3	Up	1.31	1.04	0.49
AXL	Up	0.57	1.71	0.22
C3	Up	1.90	3.58	1.92
CCL2	Up	0.38	1.82	1.14
CCL5	Up	1.22	1.82	0.77
CCNB1	Up	0.26	1.07	0.29
CD44	Up	0.99	2.66	0.67
CDC25B	Up	0.57	0.86	0.26
COL1A2	Up	1.51	3.11	1.21
COL3A1	Up	1.86	3.32	1.80
CSF2RB	Up	1.31	2.16	0.87
CXCL1	Up	0.69	1.67	0.77
CXCL12	Up	0.84	1.40	0.65
CYP1B1	Up	0.91	1.37	0.24
FAS	Up	0.35	1.51	0.70
FGF2	Up	0.57	0.01	0.22
FN1	Up	1.08	2.42	0.45
GMFG	Up	0.88	1.20	0.75
ICAM1	Up	0.11	1.17	0.27
IFI16	Up	0.63	2.25	1.09
IGFBP6	Up	1.14	1.09	0.71
IL32	Up	0.69	0.68	1.34
IL7	Up	0.79	0.87	0.58
ITGA2	Up	0.03	0.62	0.38
LCP1	Up	1.37	0.26	0.50
MARCKS	Up	0.48	2.20	0.69
MMP1	Up	1.61	0.30	0.24
MMP2	Up	1.09	0.52	0.34
NDN	Up	0.63	0.64	0.19
PEA15	Up	0.60	0.81	0.30
PECAM1	Up	0.54	0.80	0.71
RAB31	Up	0.47	1.27	0.50
RRAS	Up	0.56	0.72	0.57
S100A11	Up	0.83	0.62	1.03
SELPLG	Up	1.28	1.07	0.13
SERPINE2	Up	0.19	1.04	1.09
SPP1	Up	0.35	1.00	0.45
STAT1	Up	0.48	1.34	0.75
TES	Up	0.43	1.12	0.73
THBS1	Up	0.08	1.86	0.52
TNFAIP2	Up	0.81	1.47	0.44
TNFRSF11B	Up	0.47	1.31	1.11
TNFRSF1B	Up	0.70	0.75	0.41
TP53	Up	0.63	0.43	0.29
VIM	Up	1.14	2.88	1.40
EGF	Down	−1.91	−2.23	−1.61
EGR1	Down	−4.03	−0.68	−0.78
ESM1	Down	−1.81	−0.61	−0.94
IGFBP1	Down	−2.04	−0.93	−0.32
IQGAP2	Down	−0.68	−0.31	−0.39

Gene	Direction	logFC GSE142025	logFC GSE30529	logFC GSE104954
RHOB	Down	−0.59	−0.43	−0.63
SMPD1	Down	−0.28	−0.46	−0.26
VEGFA	Down	−1.01	−0.71	−0.72

Table S2. Sensitivity of the prioritization to leaving out each cohort and to an up-regulated-genes-only query. ρ = Spearman correlation of per-compound reversal scores with the full consensus analysis; all ten Table 3 compounds remained reversers in every variant. The up-regulated-genes-only query used the 1224 up-regulated genes of the consensus signature with an empty down-regulated set. The top-50 reverser count uses all named compounds; top-100 retention is computed within the 867-compound candidate set.

Variant	Spearman ρ	Top-50 still reversers	Top-50 within variant top-100
Leave out GSE142025	0.82	50/50	33/50
Leave out GSE30529	0.85	50/50	43/50
Leave out GSE104954	0.87	50/50	47/50
Up-regulated genes only	0.81	50/50	40/50

Table S3. Senescence gene-set coverage per cohort. Number and percentage of gene-set members present in each processed expression matrix, matched by gene symbol without alias resolution. SAUL_SEN_MAYO and FRIDMAN_SENESCENCE_UP are the sets scored by ssGSEA; FRIDMAN_SENESCENCE_DN is used only for signature construction. Coverage is lowest in GSE142025, whose deposited matrix excludes transcripts with fewer than 100 reads across all samples; the absent SAUL_SEN_MAYO members are predominantly low-abundance secreted SASP factors, and CDKN2A is also absent from that matrix. For FRIDMAN_SENESCENCE_DN, which is used only for signature construction, coverage is lowest in GSE104954 (9/13).

Gene set	Members	GSE142025	GSE30529	GSE104954
SAUL_SEN_MAYO	124	103 (83.1%)	121 (97.6%)	120 (96.8%)
FRIDMAN_SENESCENCE_UP	77	70 (90.9%)	73 (94.8%)	73 (94.8%)
FRIDMAN_SENESCENCE_DN	13	11 (84.6%)	11 (84.6%)	9 (69.2%)
